# The fraction of sensitization among lung transplant recipients in a transplant center in Japan

**DOI:** 10.1186/s12890-020-01299-0

**Published:** 2020-10-02

**Authors:** Sakiko Kumata, Takashi Hirama, Yui Watanabe, Hisashi Oishi, Hiromichi Niikawa, Miki Akiba, Jussi Tikkanen, Yoshinori Okada

**Affiliations:** 1 Department of Thoracic Surgery, Institute of Development, Aging and Cancer, Tohoku University, Sendai, Miyagi Japan; 2 Division of Organ Transplantation, Tohoku University Hospital, 4-1 Seiryomachi, Sendai, Miyagi 980-8574 Japan; 3 Multi-Organ Transplant Program, Toronto General Hospital, Toronto, ON Canada

**Keywords:** Lung transplant, Panel-reactive assay (PRA), Donor-specific antibodies (DSA), Japan, Human leukocyte antigen (HLA), Chronic lung allograft dysfunction (CLAD)

## Abstract

**Background:**

Anti-human leukocyte antigen (HLA) antibody testing was approved by the Japanese government in 2018. As such, there was no longitudinal data regarding the HLA-sensitization of lung transplant (LTX) patients in Japan. We therefore set out to measure anti-HLA antibodies from all our LTX patients during their annual follow-up to characterize the sensitization status in the Japanese population.

**Methods:**

The cross-sectional study was conducted for consecutive LTX recipients who underwent transplantation from January 2000 to January 2020 at Tohoku University Hospital (TUH). The serum from the recipients was screened for anti-HLA antibody with the panel-reactive assay (PRA) and the donor-specific antibodies (DSA).

**Results:**

Sensitization was reviewed in 93 LTX recipients, showing 23 positive (24.7%) and 70 negative (75.3%) PRA. More sensitized recipients were found in recent transplantations (60.9% (14/23), ≤5 years post LTX) than in older transplantations (17.4% (4/23), 5–10 years or 21.7% (5/23), ≥10 years post LTX) (*p* = 0.04). Even fewer recipients had DSA (5.4%, 5/93), among whom 4/5 (80%) were recently transplanted.

**Conclusion:**

The rate of PRA positive LTX recipients in our population was lower compared with those in previous reports from US and Europe. More sensitized LTRs were found in recent transplantations than the older cohort, and DSA was identified primarily in the recent recipients. Due to several limitations, it is still unclear whether the sensitization would be related the development of CLAD or survival, yet this study would be fundamental to the future anti-HLA body study in Japanese population.

## Backgrond

Japan has taken steps towards increasing number of donors and recipients and lung transplantation (LTX) has become a standard clinical practice for patients with advanced respiratory disease. Due to severe shortage of donor organs, the country has a unique profile in transplantation [1] such that the number of unilateral LTX outnumbers that of the bilateral. In addition, an upper age limit has been set for the registry of transplantation (younger than 60 years old for the unilateral transplant and 55 for the bilateral) and living-donor lobar LTX is considered an ethically justifiable procedure and still accounts for 10–20% of transplants [1].

The role of donor-specific antibodies (DSA) against class I and II human leukocyte antigen (HLA) has gained increasing attention in LTX. Development of DSA is associated with increased chronic lung allograft dysfunction (CLAD) and morbidity and mortality in LTX recipients [2–4]. In Japan, there is no data regarding the HLA-sensitization of LTX patients until 2018 as anti-HLA antibody testing was not covered by the government. Therefore, there is no existing knowledge on the postoperative sensitization status of LTX recipients in Japan.

In this study, we set out to serially measure anti-HLA antibodies from all our LTX patients during their yearly follow-up to characterize the sensitization status in our post LTX population.

## Methods

### Study design and data collection

All LTX recipients (LTR) who underwent transplantation from January 2000 to January 2020 at Tohoku University Hospital (TUH) were consecutively included in the analysis. The cross-sectional study was conducted between January 2019 and January 2020, when the anti-HLA antibody was measured in LTRs who visited the annual assessment. LTRs were fully assessed at months 3 and 6 post-transplant and thereafter annually. Participants’ clinical details at the time of transplant were reviewed from the medical records, and the current status of lung function was assessed when the blood sample was collected. The study protocol was reviewed by the Institutional Review Board (IRB) at Tohoku University Hospital (IRB number 2019-01-54). In light of the retrospective design, the requirement of informed consent was waived. We disclosed information on the implementation of the research and ensured the opportunity for research subjects to refuse participation by posting the information disclosure materials approved by the Ethics Committee on the website of the Graduate School of Medicine, Tohoku University.

### Anti-HLA antibody test

The serum from the recipients was screened for anti-HLA antibody with Flow PRA (One Lambda, Inc., Canoga Park, CA). The LTR was considered sensitized when the panel-reactive assay (PRA) was positive (greater than 0%) and regarded unsensitized when PRA was negative. The sensitized recipients were further tested for donor-specific antibody (DSA) with LABScreen single-antigen beads (One Lambda, Inc.). The positive DSA was defined by mean fluorescence intensity (MFI) units greater than 1000 or more. The complement-binding function in DSA was measured with C1q Screen (One Lambda Inc.). The test for anti-HLA antibody was done in ReproCELL Japan Inc. Yokohama.

### HLA testing in Japan

Pre-transplant PRA was not covered by the government and not routinely measured in LTX candidates so far. At the time of transplant, HLA typing at A, B and DRB1 loci was measured in both a donor and recipients, with additional loci typing in our center since 2018. Prior to transplant performed, complement dependent cytotoxicity (CDC) T cell cross-match using current or historic sera needs to be carried out without dithiothreitol (DTT) reduction, which is applied for transplant decision-making; the candidates must be negative in CDC cross-match. For the time being, CDC B cell cross-match and flow cytometry cross-match are not routinely performed in Japan. As HLA typing at HLA-C and DQB1 in donors has not been measured until 2018, Cw- and DQ-DSA were not fully assessed in this cohort.

### Immunosuppression protocol and acute allograft rejection

Induction of immunosuppression was achieved using basiliximab. Maintenance of immunosuppression consists of tacrolimus targeting the trough level of 10–14 ng/ml for the first 6 months, 9–13 ng/ml up to 12 months and 8–10 ng/ml thereafter, mycophenolic acid with 1000 mg for < 50 kg or 1500 mg ≥ 50 kg as tolerated and prednisone 1.0 mg/kg for the first 4 days, followed by 0.8 mg/kg for 4 days and gradually tapered to 5 mg. When LTRs could not tolerate tacrolimus or mycophenolic acid, cyclosporine or azathioprine is the alternative, respectively. Acute allograft rejection, considered when acutely dropped lung function without episodes of infection or mechanical factors such as airway stenosis, pleural effusion or native lung hyperinflation, was treated with bolus of methylprednisolone 500 mg for 3 consecutive days, followed by tapering doses of prednisone back to 5 mg. Surveillance bronchoscopy was not routinely scheduled at TUH.

### Definition of CLAD

Chronic lung allograft dysfunction (CLAD) was defined as a substantial (≥20%) and persistent (≥3 months) decline in FEV1 from the baseline which is the mean of the best 2 post-operative FEV1 (taken > 3 weeks apart) in a lung transplant recipient (LTR) survived ≥3 months [5].

### Definition of CLAD subtypes

Restrictive allograft syndrome (RAS) was defined by a concomitant (≥10%) decline in TLC from the baseline which is the mean of the best 2 post-operative TLC (taken > 3 weeks apart) and persistent opacities on thoracic CT among those who developed CLAD [6]. Bronchiolitis obliterans syndrome (BOS) is defined by the presence of the airflow limitation (FEV1/FVC ratio < 0.70) among those who developed CLAD. Mixed phenotype is defined by both features of RAS (≥10% decline of TLC) and BOS (FEV1/FVC ratio < 0.70) among LTRs with CLAD. Undefined is considered when phenotype of CLAD is difficult to be categorized among patients with CLAD [5].

### Statistical analysis

Categoric variables were reported as percentages, and continuous variables as medians (interquartile range (IQR)) as appropriate. Differences between groups were assessed with chi-square or Fisher’s exact tests for categoric variables and Mann Whitney test (two groups) or Kruskal-Wallis (three groups) as appropriate for continuous variables. Statistical analyses were performed using EZR (Saitama Medical Center, Jichi Medical University, Omiya, Japan), which is a graphical user interface for R (The R Foundation for Statistical Computing, Vienna, Austria), where a two-sided *P* value was calculated. Graph generation was performed with GraphPad Prism 6.0 (GraphPad Software, Inc., La Jolla, CA).

## Results

**Fig. 1:**
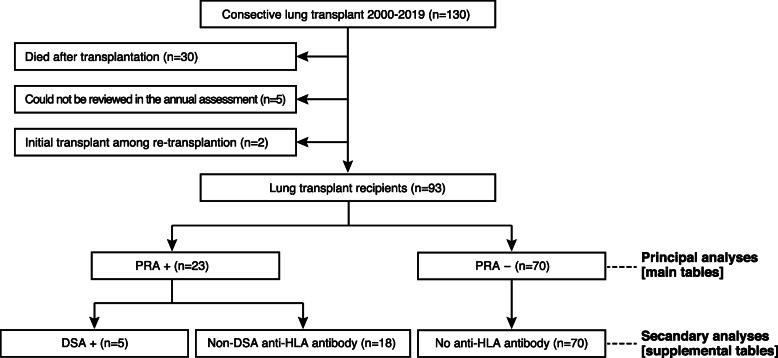
Study population

Up until January 2020, 760 LTX comprising 526 deceased-donor and 234 living-donor have been performed in Japan, of which TUH accounted for 17.1% (130/760). Of those, LTRs who have passed away (*n* = 30) and could not participated in the annual assessment (*n* = 5) were excluded from the study. The initial transplantation in re-transplanted recipients were also excluded (*n* = 2) (Fig. 1). Sensitization was then reviewed in the rest of 93 patients, showing 23 positive (24.7%) and 70 negative (75.3%) PRA. The principal analyses were to compare PRA+ (*n* = 23) with PRA- recipients (*n* = 70), summarized in the main tables. The secondary analyses were, although the number was small, to see recipients who developed the donor-specific antibody (DSA)(*n* = 5), carried non-DSA anti-HLA antibody (*n* = 18) and did not possess anti-HLA antibody (*n* = 70), summarized in supplemental files.

The patients’ characteristics at the time of transplantation were summarized (Table 1). The median age in LTRs at the time of transplantation was 42 (IQR 32–50), and 57.0% (53/93) were female (Table 1). At THU, the single transplant was the major LTX procedure at 51.6% (48/93). The obstructive lung disease was the most responsible for the LTX indication at 41.9% (39/93), followed by pulmonary vascular disease at 22.6% (21/93) and restrictive lung disease at 20.4% (19/93). There was no statistic difference in age, gender, LTX procedure and LTX indication between PRA+ and PRA- LTRs (*p* = 0.423, 1.000, 0.935 and 0.282, respectively). Pre-transplant comorbidities including diabetes (hemoglobin A1c > 6.5%), connective tissue disease, chronic kidney disease (the glomerular filtration rate < 60 mL/min/1.73 m2) [7] and gastroesophageal reflux disease (typical symptoms and endoscopic changes) [8], were also assessed in PRA+ and PRA- groups without significance (*p* = 1.000, 0.100, 0.590 and 1.000, respectively). In secondary analyses, no statistic difference was found in age, gender, LTX procedure, LTX indication and pre-transplant comorbidities among DSA+, non-DSA anti-HLA antibody and PRA- recipients (Supplemental Table 1).

**Table 1:** Patients’ characteristics at the time of transplant in the study (*n* = 93) and lung transplant recipients with/without PRA (*n* = 23 vs 70, respectively)

	Total (***n*** = 93)	PRA+ (***n*** = 23)	PRA- (***n*** = 70)	*p*-value
**Age at LTX, median (IQR)**	42 (32–50)	45 (33–50)	41 (31–49)	0.423
**Sex, female**	53 (57.0%)	13 (56.5%)	40 (75.5%)	1.000
**LTX procedure**				
**Single**	48 (51.6%)	11 (47.8%)	37 (52.9%)	0.935
**Double**	37 (39.8%)	10 (43.5%)	27 (38.6%)	
**Living-donor**	8 (8.6%)	2 (8.7%)	6 (8.6%)	
**LTX indication**				
**Pulmonary vascular disease**	21 (22.6%)	4 (17.4%)	17 (24.3%)	0.282
**Restrictive lung disease**	19 (20.4%)	7 (30.4%)	12 (17.1%)	
**Obstructive lung disease**	39 (41.9%)	7 (30.4%)	32 (45.7%)	
**Suppurative lung disease**	8 (8.6%)	4 (17.4%)	4 (5.7%)	
**CLAD**	2 (2.2%)	0 (0%)	2 (2.9%)	
**Others**	4 (4.3%)	1 (4.3%)	3 (4.3%)	
**Pre-LTX comorbidities**				
**Diabetes**	6 (6.5%)	1 (4.3%)	5 (7.1%)	1.000
**Connective tissue disease**	14 (15.1%)	6 (23.1%)	8 (11.4%)	0.102
**GERD**	5 (12.8%)	2 (8.7%)	3 (4.3%)	0.594
**Chronic kidney disease**^**a**^	3 (3.3%)	1 (4.3%)	2 (2.9%)	1.000

Abbreviation: *PRA* panel-reactive assay, *IQR* Interquartile range, *LTX* lung transplant, *CLAD* chronic lung allograft dysfunction, *GERD* gastroesophageal reflux disease

^a^incalculable the glomerular filtration rate in 2 recipients (*n* = 91)

The transplant surgery was then reviewed (Table 2). Donor age (≤19, 20–59, ≥60 years old), operative time (≤419, 420–839, ≥840 min) and total ischemic time (≤419, 420–599, ≥600 min) were partitioned and compared in PRA+ and PRA- groups, without statistic difference (*p* = 0.09, 0.900 and 0.861, respectively). CMV mismatch was seen less in PRA+ (4.3%) than in PRA- recipients (21.4%) without significance (*p* = 0.06). Recently transplanted recipients (≤5 years post LTX) had a higher proportion of positive PRA (60.9% (14/23)) compared to patients transplanted in earlier eras with 17.4% (4/23) in 5–10 years and 21.7% (5/23) in ≥10 years post LTX (*p* = 0.04). In secondary analyses, altogether 5 patients had DSA, 4/5 (80%) were recently transplanted while the remaining patient (20%) was 5–10 years post LTX. There was no positive DSA in in recipients seen ≥10 years post LTX (Supplemental Table 2).

**Table 2:** Transplant surgery and years since transplant in the study (*n* = 93) and lung transplant recipients with/without PRA (*n* = 23 vs 70, respectively)

	Total (***n*** = 93)	PRA+ (***n*** = 23)	PRA- (***n*** = 70)	*p*-value
**CMV mismatch (D+/R-)**^**a**^	16 (17.2%)	1 (4.3%)	15 (21.4%)	0.058
**Donor age$**				0.110
**≤ 19**	9 (9.7%)	0 (0%)	9 (12.9%)	
**20–59**	79 (84.9%)	20 (87.0%)	59 (84.3%)	
**≥ 60**	4 (4.3%)	2 (8.7%)	2 (2.9%)	
**Operative time (min)**				0.868
**≤ 419**	24 (25.8%)	5 (21.7%)	19 (27.1%)	
**420–839**	47 (50.5%)	12 (52.2%)	35 (50.0%)	
**≥ 840**	22 (23.7%)	6 (26.1%)	16 (22.9%)	
**Ischemic time (min)**^**b**^				0.861
**≤ 419**	21 (22.6%)	5 (21.7%)	16 (22.9%)	
**420–599**	42 (45.2%)	9 (39.1%)	33 (47.1%)	
**≥ 600**	29 (31.2%)	8 (34.8%)	21 (30.0%)	
**Yeas since transplant**				0.048
**≤ 5 years**	40 (43.0%)	14 (60.9%)	26 (37.1%)	
**5–10 years**	36 (38.7%)	4 (17.4%)	32 (45.7%)	
**≥ 10 years**	17 (18.3%)	5 (21.7%)	12 (17.1%)	

Abbreviation: *PRA* panel-reactive assay, *CMV* cytomegalovirus, *D* donor, *R* recipient

^a^missing the CMV serology in 17 recipients (*n* = 76), $missing the age in one donor (*n* = 92) and ^b^missing the ischemic time in one recipient (*n* = 92)

From the standpoint of HLA sensitization, pregnancy, transfusion and prior transplant are generally considered risk factors [9, 10]. Thus, a history of pregnancy, blood transfusion and prior transplant were reviewed. This revealed no association of these factors with positive PRA (*p* = 0.280, 0.443 and 0.331, respectively) (Table 3). Additionally, there was no difference in those episodes for the development of DSA (Supplemental Table 3). On the other hand, HLA-A, B and DR alleles in both recipient and donor(s) were available for analysis in 53 LTX cases, and the number of HLA mismatch was counted. The 0–2 mismatch (0–2/6 in deceased-donor or 0–4/12 in living-donor) was found in 7 LTRs, of whom 6 (85.7%) were living-donor transplants. Meanwhile, positive DSA was seen only in the 5–6 HLA mismatch. Yet, there was no significance in the number of HLA mismatches among PRA+/− recipients (*p* = 0.796) (Table 3) as well as among DSA+, non-DSA anti-HLA antibody and PRA- recipients (*p* = 0.509) (Supplemental Table 3).

**Table 3:** Possible risk factors of sensitization (*n* = 93) and HLA mismatch (*n* = 53) among lung transplant recipients with/without PRA

	**Total (*n*** **= 93)**	**PRA+ (*n*** **= 23)**	**PRA- (*n*** **= 70)**	*p*-value
**History of pregnant**	24 (25.8%)	8 (34.8%)	16 (22.9%)	0.280
**History of transfusion**	84 (90.3%)	22 (95.7%)	62 (88.6%)	0.443
**Prior transplant**^**a**^	6 (6.5%)	0 (0.0%)	6 (8.6%)	0.331
**Any episodes of acute rejection**	13 (14.0%)	2 (8.7%)	11 (15.7%)	0.508
	**Total (*n*** **= 53)**	**PRA+ (*n*** **= 13)**	**PRA- (*n*** **= 40)**	*p*-value
**HLA mismatch A/B/DR**^**b**^				0.796
**mismatch 0–2**	7 (13.2%)	1 (7.7%)	6 (15.0%)	
**mismatch 3–4**	23 (43.4%)	6 (46.2%)	17 (42.5%)	
**mismatch 5–6**	23 (43.4%)	6 (46.2%)	17 (42.5%)	

Abbreviation: *PRA* panel-reactive assay, *HLA* human leukocyte antigen

^a^including solid organ transplant and hematopoietic stem cell transplantation prior to lung transplantation

^b^the number of mismatched HLA-A/B/DR alleles (0–6) between donors and recipient in deceased-donor transplant and the total number of mismatch alleles (0–12) were divided to half in living-donor transplant

The lung function was assessed while the annual assessment in LTRs who survived at least 1-year post transplant (*n* = 84) (Table 4). CLAD was found in 20.2% (17/84) of the recipients, among whom 11.8% (2/17) were PRA+ and 88.2% (15/17) were PRA- (*p* = 0.190). RAS accounted for 29.4% (5/17), BOS for 41.1% (7/17), mixed phenotype for 23.5% (4/17) and unclassified for 5.9% (1/17) (data not shown). In secondary analyses, no recipients diagnosed with CLAD had DSA. Antibodies in 2 sensitized recipients who were diagnosed with CLAD were non-DSA anti-HLA antibodies (Supplemental Table 4).

**Table 4:** The association of CLAD with sensitization in those who survived one year after lung transplant (*n* = 84)

	Total (***n*** = 84)	CLAD- (***n*** = 67)	CLAD+ (***n*** = 17)	*p*-value
**PRA-**	64 (76.2%)	49 (73.1%)	15 (88.2%)	0.191
**PRA+**	20 (23.8%)	18 (26.9%)	2 (11.8%)	

Abbreviation: *PRA* panel-reactive assay, *CLAD* chronic lung allograft dysfunction

The detail of PRA and DSA in sensitized patients (*n* = 23) were summarized (Table 5). Of 23 participants, the number of LTRs with sensitization only to HLA class 1 was 9 (39.1%), of whom 3 were highly sensitized (> 25%) [11, 12] and 2 have DSA (Cw4 and B52/B59). On the other hand, the number of recipients who had only anti-HLA antibodies class 2 were 5 (21.7%), of whom no one showed PRA over 25% but one recipient had DSA (DR4). Nine sensitized LTRs with anti-HLA antibodies against both HLA class 1 and 2 (39.1%) were found, among whom 7 were highly sensitized and 2 had DSA (DR4 and DQ9). No DSA found in the study period had C1q-binding activities.

**Table 5:** The detail of PRA and DSA in the study (*n* = 93)

		DSA		
		Identification	MFI	C1q-binding
**PRA class 1+/class2-**	**9**			
PRA > 25%	3			
Non-DSA anti-HLA antibodies	7			
DSA (MFI > 1000)	2	Cw4	1243	negative
		B52, B59	4249, 2860	negative
**PRA class 1−/class2+**	**5**			
PRA > 25%	0			
Non-DSA anti-HLA antibodies	4			
DSA (MFI > 1000)	1	DR4	1248	negative
**PRA class 1+/class2+**	**9**			
PRA > 25%	7			
Non-DSA anti-HLA antibodies	7			
DSA (MFI > 1000)	2	DR4	1137	negative
		DQ9	4091	negative

Highly sensitized PRA was defined over 25%

Abbreviation: *PRA* panel-reactive antibody, *DSA* donor-specific antibody, *MFI* mean fluorescence intensity

## Discussion

The status of HLA-sensitization post transplantation was reviewed in a lung transplant center in Japan. Somewhat surprisingly, only 24.7% (23/93) Japanese recipients were PRA positive. Even fewer recipients had DSA (5.4%, 5/93), which was far lower than that of other transplant facilities, e.g. over 80% of 320 lung transplant recipients were sensitized and around a half had developed DSA within 2 years post-transplantation in Toronto, Canada [2], 50.1% (105/206) of recipients transplanted within a year had DSA in Suresnes, France [3] and 63.9% (124/194) in LTRs developed DSA in Dallas, United States [13]. However, the data shown in this study is a snapshot of a single and often remote time point from the LTX operation. Therefore, careful attention should be paid in interpreting our result with low prevalence of sensitization and DSA in our cohort.

Throughout the study, more sensitized LTRs were found in recent transplantations (≤5 years post LTX) than the earlier (5–10 years or ≥ 10 years post LTX), and the majority of recipients who carried DSA were the recent cases but not in the older ones. This can be possible survival bias that some of earlier recipients may have died owing to graft failure without ever knowing about DSA and we thus have seen less sensitized recipients in the older cohort. To further elucidate the possible bias, the cause of death was reviewed among those who died after transplant (*n* = 30) (Supplemental Table 5). Of those, 24 were found with an obvious cause of death which was highly unlikely to be related to antibody-mediated rejection (AMR). As transplantation was not scheduled when there was a positive CDC crossmatch, humoral rejection due to pre-existing DSA (hyper-acute rejection) was also unlikely in cases with primary graft dysfunction. Presence of DSA could not be denied in 6 recipients, of whom 3 CLAD cases and 1 unknown were transplanted in the earlier (> 5 years). Even in the scenario where those 4 LTRs may have died with AMR, the influence of DSA to death is still small in those population. Given the low incidence of sensitization, long survival and little impact of DSA to death, it is conceivable that the earlier recipients were less sensitized, with which the survival bias may be implausible. Instead, this raised the assumption that positive PRA or DSA could be higher in the recent cases but not in the older cohort in the population.

This could be explained if recipients surviving > 5 years post LTX might have transiently developed DSA that disappeared thereafter and are currently recognized as unsensitized. The study done in Houston, United States [14] indicated that transient de novo DSA was commonly seen and associated with a lower risk of post-LTX acute rejection than that of persistent de novo DSA. Similarly, the study from Munich, Germany [15] showed that persistent DSA was associated with reduced survival compared with transient DSA. Given these facts, our earlier cases might have had transient DSA and currently undetectable DSA, and thus show longer survival. However, it is unclear from the study design whether LTRs in our center have had DSA before or after transplant since PRA and DSA are time-dependent variables. To make those above questions more clear, a longitudinal study with repeated measurement of PRA and DSA before and after transplantation is needed. Yet, this study should be fundamental for future trials in Japan as it underlines our need to better understand our LTX population.

Based on the National Marrow Donor Program [16], HLA allele frequency varies by race and ethnicity in the US Population. With those features in mind, it should be interesting to evaluate the status of sensitization and DSA frequency in the Japanese population with a large-scale analysis as they have relatively little genetic diversity, with the notable exception of the Okinawa region [17]. In our center, the 0–2 HLA mismatch was only seen in living-donor LTX recipients who were PRA negative (Table 3), while positive DSA was seen only in the 5–6 HLA mismatch. However, it is inconclusive whether the number of mismatches is associated with HLA-sensitization as the sample size was too small to show statistical difference in the study. Meanwhile, it is worthwhile to see the HLA-DQ mismatches in the population that drives de novo DSA in LTX [18]. Despite our data, de novo DSA are common after LTX and associated with an increased risk of CLAD. However, HLA typing at DQ loci is not mandatory for transplant matching for the time being and has not been routinely investigated in Japan. To pursue the role of DQ matching between a recipient and donors, consecutive measurement of HLA loci will be required in the next study.

Given the cross-sectional analysis in a single-center, we have several limitations that warrant discussion. First, the number of LTX recipients is still in short for analysis. Despite the transplant circumstance changing in Japan, the shortage of organ donors is one of the largest issues in all solid organ transplantation [1–19]. Analysis was done in sensitized vs non-sensitized recipients, rather than positive vs negative DSA, due to the small sample number. To overcome this, the multicenter study would benefit from seeing the impact of DSA in Japanese population. Second, a universal health care insurance system in Japan began to cover the fee of anti-HLA antibody among LTX recipients in April 2018. Thus, little has been known about the status of sensitization in LTX candidates when registered and that of DSA in LTRs throughout the country. The successive measurement of DSA in LTR could be a fundamental data to analyze the relationship of de novo DSA to CLAD. Although some limitations are need to be addressed, this is the first report documenting the sensitization status and DSA in LTX recipients, so that provides valuable tip to other transplant centers in Japan.

## Conclusions

To sum up, the rate of PRA positive LTRs in the Japanese population was lower compared with those in previous reports from US and Europe. More sensitized LTRs were found in recent transplantations than the older cohort, and DSA was identified in the recent recipients. Due to several limitations, it is still unclear whether the sensitization would be related to the development of CLAD or survival, yet this study would be fundamental to the future anti-HLA body study in Japanese population.

## Supplementary information

**Additional file 1: Supplemental Table 1.** Patients’ characteristics at the time of transplant and lung transplant recipients with/without DSA (*n* = 93). **Supplemental Table 2.** Transplant surgery and years since transplant in lung transplant recipients with/without DSA (*n* = 93). **Supplemental Table 3.** Possible risk factors of sensitization (*n* = 93) and HLA mismatch (*n* = 53). **Supplemental Table 4.** The association of CLAD with/without DSA in those who survived one year after lung transplant (*n* = 84). **Supplemental Table 5.** Cause of death and possible antibody mediated rejection among transplant patients who have passed away (*n* = 30).

## Data Availability

The datasets used and/or analysed during the current study are available from the corresponding author on reasonable request.

## Abbreviations

BE: Bronchiectasis
BOS: Bronchiolitis obliterans syndrome
CLAD: Chronic lung allograft rejection
COPD: Chronic obstructive pulmonary disease
CKD: Chronic kidney disease
IPF: Idiopathic pulmonary fibrosis
IP: Interstitial pneumonia
LTX: Lung transplant
LTR: Lung transplant recipient
PAH: Pulmonary arterial hypertension
RAS: Restrictive allograft syndrome
