# Supplementary material for: The fraction of sensitization among lung transplant recipients in a transplant center in Japan
Source: BMC Pulm Med. 2020 Oct 2;20:256. doi: 10.1186/s12890-020-01299-0 (PMC7531146; doi:10.1186/s12890-020-01299-0)
Supplement: Supplementary file 1 — Additional file 1: Supplemental Table 1. Patients’ characteristics at the time of transplant and lung transplant recipients with/without DSA (n = 93). Supplemental Table 2. Transplant surgery and years since transplant in lung transplant recipients with/without DSA (n = 93). Supplemental Table 3. Possible risk factors of sensitization (n = 93) and HLA mismatch (n = 53). Supplemental Table 4. The association of CLAD with/without DSA in those who survived one year after lung transplant (n = 84). Supplemental Table 5. Cause of death and possible antibody mediated rejection among transplant patients who have passed away (n = 30). [file 12890_2020_1299_MOESM1_ESM.docx]

| **Supplemental table 1** | |  |  |  |  |  |
| --- | --- | --- | --- | --- | --- | --- |
|  |  | **Total (n=93)** | **DSA+ (n=5)** | **Non-DSA anti-HLA ab (n=18)** | **PRA- (n=70)** | p-value |
| **Age at LTX, median (ICQ)** | | 42 (32-50) | 45 (41.5-46) | 46 (34-51.5) | 41 (31-49) | 0.585 |
| **Sex, female** | | 53 (57.0%) | 2 (40.0%) | 11 (61.1%) | 40 (57.1%) | 0.745 |
| **LTX procedure** | |  |  |  |  | 0.867 |
|  | **Single** | 48 (51.6%) | 2 (40.0%) | 9 (50.0%) | 37 (52.9%) |  |
|  | **Double** | 37 (39.8%) | 3 (60.0%) | 7 (38.9%) | 27 (38.6%) |  |
|  | **Living-donor** | 8 (8.6%) | 0 (0.0%) | 2 (11.1%) | 6 (8.6%) |  |
| **LTX indication** | |  |  |  |  | 0.579 |
|  | **Pulmonary vascular disease** | 21 (22.6%) | 2 (40.0%) | 2 (11.1%) | 17 (24.3%) |  |
|  | **Restrictive lung disease** | 19 (20.4%) | 1 (20.0%) | 6 (33.3%) | 12 (17.1%) |  |
|  | **Obstructive lung disease** | 39 (41.9%) | 1 (20.0%) | 6 (33.3%) | 32 (45.7%) |  |
|  | **Suppurative lung disease** | 8 (8.6%) | 1 (20.0%) | 3 (16.7%) | 4 (5.7%) |  |
|  | **CLAD** | 2 (2.2%) | 0 (0.0%) | 0 (0.0%) | 2 (2.9%) |  |
|  | **Others** | 4 (4.3%) | 0 (0.0%) | 1 (5.6%) | 3 (4.3%) |  |
| **Pre-LTX comorbidities** | |  |  |  |  |  |
|  | **Diabetes** | 6 (6.5%) | 0 (0.0%) | 1 (5.6%) | 5 (7.1%) | 1.000 |
|  | **Connective tissue disease** | 14 (15.1%) | 2 (40.0%) | 4 (22.2%) | 8 (11.4%) | 0.111 |
|  | **GERD** | 5 (5.4%) | 1 (20.0%) | 1 (5.6%) | 3 (4.3%) | 0.288 |
|  | **Chronic kidney disease¶** | 3 (3.2%) | 0 (0.0%) | 1 (5.6%) | 2 (2.9%) | 0.587 |

**Supplemental table 1 Patients’ characteristics at the time of transplant and lung transplant recipients with/without DSA (n=93)**

Abbreviation: DSA, donor-specific antibody; PRA, panel-reactive antibody; IQR, Interquartile range; LTX, lung transplant; CLAD, chronic lung allograft dysfunction; GERD, gastroesophageal reflux disease

¶incalculable the glomerular filtration rate in 2 recipients (n=91)

| **Supplemental table 2** | |  |  |  |  |  |
| --- | --- | --- | --- | --- | --- | --- |
|  |  | **Total (n=93)** | **DSA+ (n=5)** | **Non-DSA anti-HLA ab (n=18)** | **PRA- (n=70)** | p-value |
| **CMV mismatch (D+/R-)¶** | | 16 (17.2%) | 1 (20.0%) | 0 (0.0%) | 15 (21.4%) | 0.064 |
| **Donor age$** | |  |  |  |  | 0.222 |
|  | **≤ 19** | 9 (9.7%) | 0 (0.0%) | 0 (0.0%) | 9 (12.9%) |  |
|  | **20-59** | 79 (84.9%) | 5 (100.0%) | 15 (83.3%) | 59 (84.3%) |  |
|  | **≥ 60** | 4 (4.3%) | 0 (0.0%) | 2 (11.1%) | 2 (2.9%) |  |
| **Operative time (min)** | |  |  |  |  | 0.725 |
|  | **≤ 419** | 24 (25.8%) | 0 (0.0%) | 5 (27.8%) | 19 (27.1%) |  |
|  | **420-839** | 47 (50.5%) | 3 (60.0%) | 9 (50.0%) | 35 (50.0%) |  |
|  | **≥ 840** | 22 (23.7%) | 2 (40.0%) | 4 (22.2%) | 16 (22.9%) |  |
| **Ischemic time (min)†** | |  |  |  |  | 0.562 |
|  | **≤ 419** | 21 (22.6%) | 0 (0.0%) | 5 (27.8%) | 16 (22.9%) |  |
|  | **420-599** | 42 (45.2%) | 2 (40.0%) | 7 (38.9%) | 33 (47.1%) |  |
|  | **≥ 600** | 29 (31.2%) | 3 (60.0%) | 5 (27.8%) | 21 (30.0%) |  |
| **Yeas since transplant** | |  |  |  |  | 0.083 |
|  | **≤ 5 years** | 40 (43.0%) | 4 (80.0%) | 10 (55.6%) | 26 (37.1%) |  |
|  | **5-10 years** | 36 (38.7%) | 1 (20.0%) | 3 (16.7%) | 32 (45.7%) |  |
|  | **≥ 10 years** | 17 (18.3%) | 0 (0.0%) | 5 (27.8%) | 12 (17.1%) |  |

**Supplemental table 2 Transplant surgery and years since transplant in lung transplant recipients with/without DSA (n=93)**

Abbreviation: CMV, cytomegalovirus; D, donor; R, recipient

¶missing the CMV serology in 17 recipients (n=76), $missing the age in one donor (n=92) and †missing the ischemic time in one recipient (n=92)

| **Supplemental table 3** | |  |  |  |  |  |
| --- | --- | --- | --- | --- | --- | --- |
|  |  | **Total (n=93)** | **DSA (n=5)** | **Non-DSA anti-HLA ab (n=18)** | **No PRA (n=70)** | p-value |
| **History of pregnant** | | 24 (25.8%) | 1 (20.0%) | 7 (38.9%) | 16 (22.9%) | 0.365 |
| **History of transfusion** | | 84 (90.3%) | 5 (100.0%) | 17 (94.4%) | 62 (88.6%) | 0.809 |
| **Prior transplant¶** | | 6 (6.5%) | 0 (0.0%) | 0 (0.0%) | 6 (8.6%) | 0.349 |
| **Any episodes of acute rejection** | | 13 (14.0%) | 0 (0.0%) | 2 (11.1%) | 11 (15.7%) | 1.000 |
|  |  |  |  |  |  |  |
|  |  | **Total (n=53)** | **DSA (n=2)** | **Non-DSA anti-HLA ab (n=11)** | **No PRA (n=40)** | p-value |
| **HLA mismatch A/B/DR†** | |  |  |  |  | 0.509 |
|  | **mismatch 0-2** | 7 (13.2%) | 0 (0.0%) | 1 (9.1%) | 6 (15.0%) |  |
|  | **mismatch 3-4** | 23 (43.4%) | 0 (0.0%) | 6 (54.5%) | 17 (42.5%) |  |
|  | **mismatch 5-6** | 23 (43.4%) | 2 (100%) | 4 (36.4%) | 17 (42.5%) |  |

**Supplemental table 3 Possible risk factors of sensitization (n=93) and HLA mismatch (n=53)**

Abbreviation: PRA; panel-reactive assay, HLA; human leukocyte antigen

¶including solid organ transplant and hematopoietic stem cell transplantation prior to lung transplantation

**†**the number of mismatched HLA-A/B/DR alleles (0-6) between donors and recipient in deceased-donor transplant and the total number of mismatch alleles (0-12) were divided to half in living-donor transplant

| **Supplemental table 4** | |  |  |  |
| --- | --- | --- | --- | --- |
|  | **Total (n=84)** | **CLAD- (n=67)** | **CLAD+ (n=17)** | p-value |
| **No PRA** | 64 (76.2%) | 49 (73.1%) | 15 (88.2%) | 0.382 |
| **Non-DSA anti HLA** | 17 (20.2%) | 15 (22.4%) | 2 (11.8%) |  |
| **DSA** | 3 (3.6%) | 3 (4.5%) | 0 (0.0%) |  |

**Supplemental table 4 The association of CLAD with/without DSA in those who survived one year after lung transplant (n=84)**

Abbreviation: PRA; panel-reactive assay, CLAD, chronic lung allograft dysfunction

| **Supplemental table 5** |  |  |  |
| --- | --- | --- | --- |
| **Diagnosis** | **Number (n=30)** | **Possible antibody mediated rejection (n=6)** | |
|  |  | **≤5 years since transplant** | **>5 years since transplant** |
| Infection | 5 | 0 | 0 |
| Primary graft dysfunction | 5 | 0 | 0 |
| Airway complication | 4 | 0 | 0 |
| Chronic lung allograft dysfunction | 4 | 1 | 3 |
| Cardiac failure | 3 | 0 | 0 |
| Neuropsychological problems | 2 | 0 | 0 |
| Gastrointestinal peroration or haemorrhage | 2 | 0 | 0 |
| Thrombotic microangiopathy | 1 | 0 | 0 |
| Poor physical function | 1 | 0 | 0 |
| Malignancy | 1 | 0 | 0 |
| Unknown | 2 | 1 | 1 |

**Supplemental table 5 Cause of death and possible antibody mediated rejection among transplant patients who have passed away (n=30)**
